# Mayo MASS和R2-ISS分期系统在初诊多发性骨髓瘤患者中的预后价值验证：单中心研究

**DOI:** 10.3760/cma.j.issn.0253-2727.2023.09.008

**Published:** 2023-09

**Authors:** 影 徐, 旭星 沈, 媛媛 金, 建勇 李, 丽娟 陈, 闰 张

**Affiliations:** 南京医科大学第一附属医院，江苏省人民医院血液科，南京 210029 Department of Hematology, the First Affiliated Hospital of Nanjing Medical University, Jiangsu Province Hospital, Nanjing 210029, China

**Keywords:** 多发性骨髓瘤, Mayo分期系统, R2-ISS, 预后, Multiple myeloma, Mayo staging system, R2-ISS, Prognosis

## Abstract

**目的:**

验证Mayo MASS、R2-ISS分期系统在初诊多发性骨髓瘤（MM）患者中的预后价值。

**方法:**

纳入南京医科大学第一附属医院收治的371例初诊MM患者，胞质轻链免疫荧光结合荧光原位杂交（cIg-FISH）检测患者的细胞遗传学特征，结合患者的临床资料分析其疾病分期并评估预后。

**结果:**

R-ISS分期Ⅰ、Ⅱ、Ⅲ期患者分别有37例（10.0％）、264例（71.2％）和70例（18.8％），中位无进展生存（PFS）时间分别为37、31和14个月（*P*＝0.001），中位总生存（OS）时间分别为未达到（NR）、66个月和31个月（*P*<0.001）。Mayo MASS分期Ⅰ、Ⅱ、Ⅲ期患者分别有71例（19.1％）、140例（37.7％）和160例（43.2％），中位PFS时间分别为43、27和19个月（*P*<0.001），中位OS时间分别为NR、NR和35个月（*P*<0.001）。R2-ISS分期Ⅰ、Ⅱ、Ⅲ、Ⅳ期患者分别有23例（6.2％）、69例（18.6％）、222例（59.8％）和57例（15.4％），中位PFS时间分别为47、31、25和15个月（*P*＝0.001），中位OS时间分别为NR、NR、49个月和55个月（*P*<0.001）。

**结论:**

Mayo MASS和R2-ISS分期系统在R-ISS分期基础上纳入了1q21+，分期更完善，但两种分期系统下Ⅲ期患者比例偏高，考虑与中国MM患者1q21+发生率和ISS Ⅲ期比例较高相关。

## Abstract (en)

**Objective:**

To evaluate the prognostic value of Mayo MASS and R2-ISS staging systems in patients newly diagnosed with multiple myeloma（MM）.

**Methods:**

A total of 371 patients newly diagnosed with MM in Jiangsu Province Hospital were included in the study. Cytoplasmic light chain immunofluorescence with fluorescence in situ hybridization（cIg-FISH）was performed to detect cytogenetic abnormality. Clinical characteristics were combined to analyze the disease stage and evaluate the prognosis.

**Results:**

There were 37（10.0％）, 264（71.0％）, and 70（18.8％）patients in R-ISS stage Ⅰ, Ⅱ, and Ⅲ, respectively. The median progression-free survival（PFS）times were 37, 25, and 14 months（*P*<0.001）. The median overall survival（OS）times were not reached（NR）, 66, and 30 months（*P*<0.001）. There were 71（19.1％）, 140（37.7％）, and 160（43.2％）patients in Mayo MASS stages Ⅰ, Ⅱ, and Ⅲ, and the median PFS times periods were 43, 27, and 19 months（*P*<0.001）, and the median OS times were NR, NR, 35 months, respectively（*P*<0.001）. There were, 23（6.2％）, 69（18.6％）, 222（59.8％）, and 57（15.4％）patients in R2-ISS stages Ⅰ, Ⅱ, Ⅲ, and Ⅳ, respectively. The median PFS times were 47, 31, 25, and 15 months（*P*＝0.001）, and the median OS times were NR, NR, 49, and 55 months, respectively（*P*<0.001）.

**Conclusion:**

Based on the R-ISS staging system, Mayo MASS, and R2-ISS prognostic staging system incorporated 1q21+, which allows a better stratification. However, the proportion of stage Ⅲ patients in Mayo MASS and R2-ISS staging systems is relatively high, which is considered related to the high incidence of 1q21+ and ISS Ⅲ in the Chinese population.

多发性骨髓瘤（MM）具有高度异质性，精确的预后评估与风险分层对于MM的精准治疗至关重要。修订的国际分期系统（R-ISS）[1]是临床常用的风险分层系统，但R-ISS存在一定局限性，约62％的患者被归为R-ISS Ⅱ期，这部分患者可能有不同的生存情况。此外，有研究表明1q21+是初诊MM（NDMM）的独立不良预后因素，但R-ISS并未考虑这一因素。因此，在R-ISS分期的基础上，梅奥临床中心和欧洲骨髓瘤工作组分别提出Mayo MASS[2]和R2-ISS[3]分期系统，关于这两个分期系统是否适合中国NDMM患者的报道较少。本研究回顾性分析了南京医科大学第一附属医院371例NDMM患者，对R-ISS、Mayo MASS、R2-ISS三种分期系统进行比较，从而探讨Mayo MASS和R2-ISS分期系统在中国NDMM患者中的预后价值。

## 病例与方法

1. 病例：纳入2012年8月至2022年5月在江苏省人民医院就诊的NDMM患者371例，诊断均符合《中国多发性骨髓瘤诊治指南（2022年修订）》[4]的诊断标准。收集患者的临床资料，包括性别、年龄、分型、DS分期、ISS分期、R-ISS分期、HGB、乳酸脱氢酶（LDH）、肌酐（CRE）、β_2_-微球蛋白（β_2_-MG）等。本研究经江苏省人民医院医学研究伦理委员会批准（2022-SR-448）。

2. FISH检测：采用胞质轻链免疫荧光结合荧光原位杂交（cIg-FISH）技术进行细胞遗传学分型，Ficoll分离患者骨髓液单个核细胞，制片、固定、老化、脱水、变性、再次脱水，使用对应的探针杂交过夜。在荧光显微镜下行原始荧光图像采集，通过浆细胞计数进行分析判断[5]。利用相应的探针检测1q21+、del（17p）、t（4;14）、t（14;16）和t（11;14）。根据国际推荐的标准，上述核型异常的参考阈值：1q21+和del（17p）为20％，t（4;14）、t（14;16）和t（11;14）为10％[6]。

3. 分期标准：Mayo MASS分期系统：ISS Ⅲ期、高危IGH易位、1q21+、del（17p）和LDH升高各积1分，累计分值0分为Ⅰ期，1分为Ⅱ期，≥2分为Ⅲ期。

R2-ISS分期系统：ISS Ⅱ期、LDH升高、del（17p）和t（4;14）各积1分，ISS Ⅲ期积1.5分，1q21+积0.5分，累计分值0分为Ⅰ期，0.5～1分为Ⅱ期，1.5～2.5分为Ⅲ期，3～5分为Ⅳ期。

4. 治疗方案：371例患者均接受以新药为基础的方案诱导化疗，其中103例患者接受以硼替佐米为基础的方案，包括VAD（硼替佐米＋表阿霉素＋地塞米松）、VCD（硼替佐米＋环磷酰胺＋地塞米松）、VD（硼替佐米＋地塞米松）方案；45例接受以免疫调节剂为基础的方案，包括CTD（环磷酰胺＋沙利度胺＋地塞米松）、TAD（沙利度胺＋表阿霉素＋地塞米松）、TD（沙利度胺＋地塞米松）、RD（来那度胺＋地塞米松）、BiRD（克拉霉素＋来那度胺＋地塞米松）方案；206例接受硼替佐米联合免疫调节剂方案，包括VRD（硼替佐米＋来那度胺＋地塞米松）、VTD（硼替佐米＋沙利度胺＋地塞米松）；17例患者接受其他方案治疗。99例患者达到部分缓解（PR）及以上后接受自体造血干细胞移植。诱导治疗有效的患者均继续接受巩固维持治疗，直至疾病复发、进展或发生严重药物不良反应后更换化疗方案。

5. 疗效评估：疗效及评估标准参考2016年国际骨髓瘤工作组（IMWG）的疗效标准[7]，分为严格意义的完全缓解（sCR）、完全缓解（CR）、非常好的部分缓解（VGPR）、PR、微小缓解（MR）、疾病稳定（SD）、疾病进展（PD）。

6. 随访：通过查阅患者病历及电话进行随访，随访截止日期为2022年5月31日，中位随访时间为34（5～118）个月。总生存（OS）期定义为自患者诊断至因任何原因死亡或者随访截止的时间间隔。无进展生存（PFS）期定义为自患者诊断至复发、死亡或随访截止的时间间隔。

7. 统计学处理：采用SPSS 25.0及GraphPad Prism 8.0两种软件进行统计学分析与作图。计数资料用例数（百分比）描述，计量资料用中位数（范围）描述。采用Kaplan-Meier曲线对患者的PFS和OS进行分析，利用Log-rank法比较组间生存差异。采用双侧检验，*P*<0.05为差异有统计学意义。

## 结果

1. 临床特征：371例患者中，男215例（58.0％），女156例（42.0％），中位发病年龄63（34～84）岁，年龄≤65岁患者231例（62.3％），>65岁患者140例（37.7％）。IgG型179例（48.2％），IgA型84例（22.6％），轻链型86例（23.2％），IgD型14例（3.8％），不分泌型4例（1.1％），IgM型3例（0.8％），IgE型1例（0.3％）。LDH升高47例（12.7％）。DS分期Ⅰ期15例（4.0％），Ⅱ期55例（14.8％），Ⅲ期301例（81.2％）；ISS分期Ⅰ期45例（12.1％），Ⅱ期142例（38.3％），Ⅲ期184例（49.6％）。细胞遗传学分析结果显示，1q21+患者188例（50.7％），del（17p）患者40例（10.8％），t（4;14）患者62例（16.7％），t（14;16）患者9例（2.4％），t（11;14）患者38例（10.3％）。

2. R-ISS、Mayo MASS、R2-ISS分期情况：R-ISS分期Ⅰ期37例（10.0％），Ⅱ期264例（71.2％），Ⅲ期70例（18.8％）；Mayo MASS分期Ⅰ期71例（19.1％），Ⅱ期140例（37.7％），Ⅲ期160例（43.2％）；R2-ISS分期Ⅰ期23例（6.2％），Ⅱ期69例（18.6％），Ⅲ期222例（59.8％），Ⅳ期57例（15.4％）。R-ISS分期Ⅰ期伴1q21+患者14例（37.8％），Ⅱ期伴1q21+患者131例（49.6％），Ⅲ期伴1q21+患者43例（61.4％），三组的差异无统计学意义（*P*＝0.055）；Mayo MASS分期Ⅰ期伴1q21+患者0例，Ⅱ期伴1q21+患者58例（41.4％），Ⅲ期伴1q21+患者130例（81.3％），三组的差异有统计学意义（*P*<0.001）；R2-ISS分期Ⅰ期伴1q21+患者0例，Ⅱ期伴1q21+患者15例（21.7％），Ⅲ期伴1q21+患者123例（55.4％），Ⅳ期伴1q21+患者50例（87.7％），四组的差异有统计学意义（*P*<0.001）。接受自体造血干细胞移植患者99例（26.7％）。三种分期系统各分期诱导治疗方案的差异均无统计学意义（*P*值均>0.05）。321例患者可进行疗效评估，95例（29.6％）获得sCR，59例（18.4％）获得CR，83例（25.9％）获得VGPR，52例（16.2％）获得PR，8例（2.5％）获得MR，13例（4.0％）评估为SD，11例（3.4％）评估为PD。R-ISS、Mayo MASS、R2-ISS不同分期的患者例数详见表1。

**表1:** 371例多发性骨髓瘤患者R-ISS、Mayo MASS、R2-ISS分期情况（例）

R-ISS分期	R2-ISS分期				Mayo MASS分期		
	Ⅰ期（23例）	Ⅱ期（69例）	Ⅲ期（222例）	Ⅳ期（57例）	Ⅰ期（71例）	Ⅱ期（140例）	Ⅲ期（160例）
Ⅰ期（37例）	23	14	–	–	23	14	0
Ⅱ期（264例）	–	55	199	10	48	126	90
Ⅲ期（70例）	–	–	23	47	–	–	70

**注** –：无

R-ISS Ⅱ期患者在R2-ISS分期中有55例（20.8％）为Ⅱ期，199例（75.4％）为Ⅲ期，10例（3.8％）为Ⅳ期，三组患者OS、PFS的差异均无统计学意义（*P*值分别为0.113、0.385）。R-ISS Ⅱ期患者在Mayo MASS分期中有48例（18.2％）为Ⅰ期，126例（47.7％）为Ⅱ期，90例（34.1％）为Ⅲ期，三组患者OS、PFS的差异均有统计学意义（*P*值分别为<0.001、0.025）。

3. R-ISS、Mayo MASS、R2-ISS分期患者的生存分析：R-ISS分期Ⅰ、Ⅱ、Ⅲ期患者的中位PFS时间分别为37、25、14个月（*P*＝0.001）；中位OS时间分别为未达到（NR）、66个月、31个月（*P*<0.001）（图1）。Mayo MASS分期Ⅰ、Ⅱ、Ⅲ期患者的中位PFS时间分别为43、27、19个月（*P*<0.001）；中位OS时间分别为NR、NR、35个月（*P*<0.001）（图2）。R2-ISS分期Ⅰ、Ⅱ、Ⅲ、Ⅳ期患者的中位PFS时间分别为47、31、25、15个月（*P*＝0.001）；中位OS时间分别为NR、NR、49个月、55个月（*P*<0.001）（图3）。

**图1:**
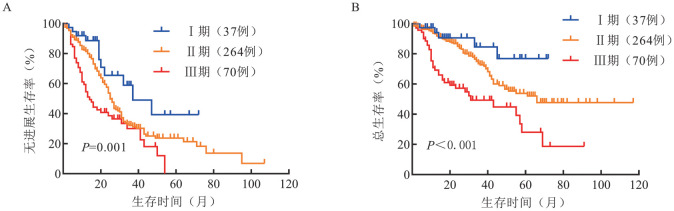
不同R-ISS分期多发性骨髓瘤患者的无进展生存（A）和总生存（B）曲线

**图2:**
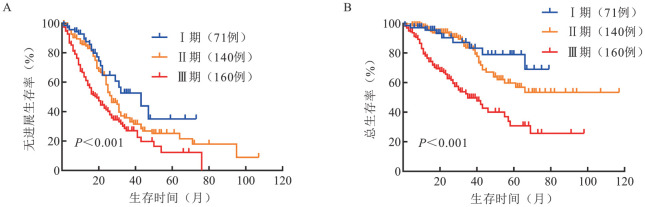
不同Mayo MASS分期多发性骨髓瘤患者的无进展生存（A）和总生存（B）曲线

**图3:**
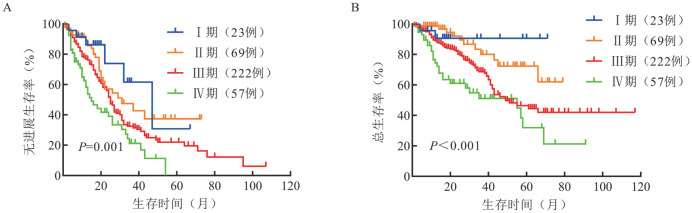
不同R2-ISS分期多发性骨髓瘤患者的无进展生存（A）和总生存（B）曲线

## 讨论

由于宿主因素、肿瘤负荷、细胞遗传学异常和对治疗的反应等多方面的差异[8]，MM患者的预后存在很大异质性，针对MM患者精确的预后评估和危险分层意义重大。传统的DS和ISS分期均未考虑细胞遗传学异常[9]，随着细胞遗传学异常在MM预后评价中的价值得到肯定，ISS分期已不能满足当前的临床需要，将细胞遗传学和LDH水平纳入预后评价指标的R-ISS分期系统在临床中得到广泛应用。但R-ISS分期存在一定的局限性，约62％的患者被归为R-ISS Ⅱ期[10]，本中心R-ISS Ⅱ期患者占69.3％，这部分患者可能有不同的生存情况，其预后的差异性被掩盖。此外，R-ISS分期虽然纳入了细胞遗传学异常，但未考虑到同时存在高危细胞遗传学异常的复合效应，影响判断预后的准确性。

1q21+是MM最常见的细胞遗传学异常，大约40％的NDMM会出现1q21+，在复发难治MM中的发生率高达70％[11]–[12]。法国骨髓瘤工作组（IFM）的临床试验显示，1 635例NDMM患者中有581例（35.5％）伴1q21+[13]。1q21+在中国人群中的比例更高，Xu等[14]的一项研究纳入358例NDMM患者，其中174例（48.6％）伴1q21+，本中心371例NDMM中，50.7％的患者伴1q21+。1q21区域包含大量基因，包括IL-6R、MCL-1、ILF2、BCL9和CKS1B，1q21+通常会导致上述基因表达失调，从而促进肿瘤细胞恶性增殖[15]。鉴于R-ISS分期对于预测预后的局限性及1q21+的高发生率，梅奥临床中心和欧洲骨髓瘤工作组分别提出了Mayo MASS和R2-ISS分期系统，不同的是，Mayo MASS将危险因素等权重纳入预后分层模型，将患者分为Mayo MASS Ⅰ期（36％）、Ⅱ期（33％）和Ⅲ期（31％）[2]。R2-ISS分期则删除对PFS无显著预后意义的t（14;16），并根据危险因素的预后权重进行赋分，将患者分为R2-ISSⅠ期（19.2％）、Ⅱ期（30.8％）、Ⅲ期（41.2％）和Ⅳ期（8.8％）[3]。

我们对本中心NDMM患者进行Mayo MASS和R2-ISS分期，发现Mayo MASS Ⅲ期（43.2％）和R2-ISS Ⅲ期（59.8％）、Ⅳ期（15.4％）患者比例均高于上述研究，可能与本中心1q21+（50.7％）和ISS Ⅲ期（49.6％）患者比例较高有关。国内的Yang等[16]和Yan等[17]也分析了Mayo MASS和R2-ISS在NDMM患者中的预后价值。Yang等[16]的队列中1q21+患者占51.8％，ISS Ⅲ期患者占52.7％，Mayo MASS Ⅲ期患者占50.3％。Yan等[17]的队列中R2-ISS Ⅲ期患者占52.3％，Ⅳ期患者占15.6％，与本中心一致，均高于梅奥临床中心和欧洲骨髓瘤工作组。本中心数据显示，Mayo MASS和R2-ISS分期能更好地对预后不良患者进行分组，但中位OS、PFS时间均较梅奥临床中心和欧洲骨髓瘤工作组的研究缩短，考虑与本中心随访时间较短（34个月、74.4个月、75.5个月）、接受移植患者比例较低（26.7％、55％、65％）有关。Yang等[16]的研究显示Mayo MASS Ⅰ、Ⅱ和Ⅲ期患者的中位PFS时间分别为45.6、27.4和20.3个月，中位OS时间分别为88.3、62.9和40.6个月。Yan等[17]的研究显示R2-ISS Ⅰ、Ⅱ、Ⅲ和Ⅳ期患者的中位PFS时间分别为76.6、64.3、33.8和30.7个月，中位OS时间分别为NR、NR、63.8个月和48.2个月，与本中心结果相近。

本中心的数据显示，三种分期系统对OS的预测效能有明显差异，Mayo MASS和R2-ISS分期对于Mayo MM患者预后的预测更为准确。此外，不同分期系统各组之间诱导治疗方案无明显差别。R-ISS分期Ⅱ期患者比例高，这部分患者的预后具有差异性，应用Mayo MASS和R2-ISS分期能更好地将其进行分组，遗憾的是，其他已被报道的独立危险因素如1p32缺失、MYC重排、微小残留病阴性、循环肿瘤细胞等未被考虑在内。中国NDMM患者的1q21+发生率和ISS Ⅲ期比例较高，使更多患者分布于MASS Ⅲ期和R2-ISS Ⅲ、Ⅳ期。本研究为单中心、回顾性研究，样本量较小，需进一步设计多中心、前瞻性研究，进一步探索适宜中国人群的预后分期系统。
